# Public–Private Mix Models of Tuberculosis Care in Pakistan: A High-Burden Country Perspective

**DOI:** 10.3389/fpubh.2021.703631

**Published:** 2021-08-10

**Authors:** Waseem Ullah, Ahmad Wali, Mahboob Ul Haq, Aashifa Yaqoob, Razia Fatima, Gul Majid Khan

**Affiliations:** ^1^Department of Pharmacy, Quaid-i-Azam University Islamabad, Islamabad, Pakistan; ^2^Department of Pharmacy Practice, Shifa Tameer-e-Millat University Islamabad, Islamabad, Pakistan; ^3^Health Department, Provincial Tuberculosis Control Program Balochistan, Quetta, Pakistan; ^4^Policy Strategy and Drug Management Unit, National Tuberculosis Control Program, Islamabad, Pakistan; ^5^Research Unit, National Tuberculosis Control Program, Islamabad, Pakistan; ^6^Islamia College University, Peshawar, Pakistan

**Keywords:** infectious disease control, healthcare system, lower middle income countries, health policy & practice, global health, public health

## Abstract

**Introduction:** Pakistan ranks fifth in the globally estimated burden of tuberculosis (TB) case incidence. Annually, a gap of 241,688 patients with TB exists between estimated TB incidence and actual TB case notification in Pakistan. These undetected/missed TB cases initiate TB care from providers in the private healthcare system who are less motivated to notify patients to the national database that leads to significant underdetection of actual TB cases in the Pakistani community. To engage these private providers in reaching out to missing TB cases, a national implementation trial of the Public–Private Mix (PPM) model was cohesively launched by National TB Control Program (NTP) Pakistan in 2014. The study aims to assess the implementation, contribution, and relative treatment outcomes of cohesively implemented PPM model in comparison to the non-PPM model.

**Methods:** A retrospective record review of all forms (new and relapse) patients with TB notified from July 2015 to June 2016 was conducted both for PPM- and non-PPM models.

**Results:** The PPM model was implemented in 92 districts in total through four different approaches and contributed 25% (81,016 TB cases) to the national TB case notification. The PPM and non-PPM case notification showed a strong statistical difference in proportions among compared variables related to gender (*p* < 0.001), age group (*p* < 0.000), and province (*p* < 0.000). Among PPM approaches, general practitioners and non-governmental-organization facilities achieve a treatment success of 94–95%; private hospitals achieve 82% success, whereas Parastatals are unable to follow more than half of their notified TB cases.

**Discussion:** The PPM model findings in Pakistan are considerably consistent with countries that have prioritized PPM for an increasing trend in the TB case notification to their national TB control programs. Different PPM approaches need to be scaled up in terms of PPM implemented districts, PPM coverage, PPM coverage efficiency, and PPM coverage outcome in the Pakistani healthcare system in the future.

## Introduction

Despite management advances in the 21st century, tuberculosis (TB) remains the leading cause of death from curable infection worldwide (1). The world population is 7.7 billion, and one-fourth (1.7 billion) of the global community is estimated to be latently infected with TB (2), which makes it the 10th leading cause of death worldwide (3). Among the infected population, Pakistan ranks fifth in the global TB burden (4). In 2019, there was an estimated number of 570,000 incident cases, an actual number of 328,312 notified cases who started treatment under national TB management guidelines, and 241,688 missed TB cases in the Pakistani community (4).

To narrow the gap between estimated and notified TB cases in high-burden countries including Pakistan, the WHO has recommended a model of TB care, which is termed as Public–Private Mix (PPM) intervention. PPM refers to engagement by the National Tuberculosis Control Program (NTP) of different countries with private sector providers of TB care in those countries (5). However, NTP Pakistan in 2005 achieved 100% coverage of WHO-recommended Directly Observed Treatment Short Course Therapy (DOTS) in all health facilities within the public sector (6). But, the PPM model was launched on a pilot scale in 2004, and this initiative was area-specific only (7).

After almost a decade of pilot-scale engagement of the PPM model in Pakistan, WHO reported that a gap of 3.3 million TB cases exists between estimated incidence and notified cases across the globe and Pakistan alone, was contributing 7% to this globally estimated gap. This was evident because the Pakistan TB case detection rate in 2013 was standing at 58% and it was missing 42% of the estimated number of incident cases (8). Viewing the undiagnosed cases with active TB that can transmit the disease into 30–50% of their extended contacts (9), missing cases burden was a public health challenge for NTP stakeholders.

To address this challenge of the under-detection of TB cases, NTP in 2014 mobilized exploratory research studies on improving the case detection rate in Pakistan. National TB prevalence survey and capture–recapture study of private-sector TB facilities in Pakistan indicated under-reporting of detected TB cases and under-diagnosis (10, 11). It was also documented that the treatment of diagnosed TB cases in the private sector may be non-adherent to NTP guidelines (12). Since the motivation of private providers to manage TB in clinics varies, so treatment attrition rates are high, and intermittent TB case management practices can lead to the emergence of drug-resistant TB cases in the Pakistani healthcare system (13). Finally, analysis of area-specific PPM implemented projects between 2004 and 2009 suggested that sustained, extended involvement of PPM approaches is required in the country followed by large-scale quantitative and qualitative studies for assessing the efficacy and cost-effectiveness of these implemented models (14).

During the ongoing exploratory research, the NTP annual report 2014 also indicated that the involvement of a private stakeholder in TB care in Pakistan is quite large and diverse. They are not only limited to formal providers like private general practitioners but also include informal providers like pharmacists, nurses, chemists, and philanthropists. This list also adds up semigovernment and large private hospitals, non-governmental organizations (NGOs), insurance agencies, community and religious leaders, researchers, and industries involved in mineral resource extraction (15).

The above list of private stakeholders and exploratory research findings triggered NTP to intensify collaboration with private providers in TB care. This led to extending the *ad hoc*-scale functioning of PPM toward the cohesive, large-scale implementation of the PPM model in 2014. The NTP team then defined the following four approaches for implementing the PPM model at the national level: solo general practitioner (GP) model, run TB care facility model of NGOs, private hospital models, and other public sector (Parastatal) models. All four approaches of PPM were categorized shortly as PPM-1, PPM-2, PPM-3, and PPM-4, respectively (15).

Once adopted in 2014, overall, the contribution of PPM intervention to TB case notifications and outcomes is known; however, the individual contribution of different PPM approaches at a large scale is not documented. Given the varied TB stakeholders in the Pakistani arena, testing which PPM models contribute the most and which models work in particular geographic settings is important; these data can help in national TB-related program planning. Based on this rationale, we designed a study to assess the contribution of four approaches of the cohesively implemented PPM model toward the national TB case notification. The objectives were specifically to explore the implementation mechanism of different PPM approaches in all four Pakistani provinces, to determine the proportion of TB cases notified by the implemented PPM approaches (private sector) in comparison to the non-PPM model (public sector) in program data of NTP, and to compare the relative treatment outcomes of PPM-notified cases to non-PPM TB cases.

## Materials and Methods

### Study Design

A descriptive cross-sectional study based on the retrospective review of routinely collected NTP data from July 2015 to June 2016.

### Study Context

All four provinces (i.e., Punjab, Sindh, Khyber Pakhtunkhwa, and Balochistan) of Pakistan. A total of 122 districts (36 in Punjab, 29 in Sindh, 25 in Khyber Pakhtunkhwa, and 32 in Balochistan) are included in these four provinces (16), and almost 97% of the Pakistani population resides in this geographical area (17). The PPM model has been cohesively implemented in these four provinces since 2014.

### Study Population

All forms of patients (new and relapse cases) with TB are notified in PPM- and non-PPM-implemented districts.

### Ethical Approval

Ethical approval for conducting this project was obtained from the Advanced Studies and Research Board, Quaid-i-Azam University Islamabad, and the Institutional Review Board (IRB) Ethics Committee, Research Unit, NTP, Ministry of National Services, Regulations, and Coordination, Islamabad, Pakistan (F.1-7/MISC-2017/151).

### Data Collection

The PPM implementation mechanism and PPM participating facilities in the operational districts were abstracted from the NTP database. The NTP support mechanism (material supply, financial support, and technical support) for the PPM model implementation was also checked during the study period.

Aggregate data of notified TB cases and their relative treatment outcomes were extracted from the quarterly case notification report form (TB-07) and quarterly outcome report form (TB-09), respectively, for PPM and non-PPM models. The data of the patient reported to NTP by the participating TB facilities are compiled in the TB-07 and TB-09 forms. All patients with TB-07 form have a TB-09 one. TB-07 categorizes the patient demographics like gender, age, province, type of patient, and disease classification, whereas TB-09 records the outcome of the patient notified on TB-07. These two forms are updated quarter-wise, and a report is generated in the form of Microsoft Excel spreadsheets (Microsoft Corp., WA, USA) in each NTP implemented district (district level). These quarterly reports are then compiled at the provincial level and finally at the national level. So, data were compiled for all four quarterly national spreadsheets from mid-2015 to mid-2016.

Data collection was performed on different time periods, both for TB case notification and treatment outcomes of the notified cases. This is because a TB case (PPM and non-PPM) once notified at TB facility on TB-07 form, then the treatment outcome of that notified case is recorded on TB-09 after 6–9 months of initiating drug-susceptible TB treatment (i.e., by the end or after third NTP quarterly review meeting from the date of notifying TB case), which is then finally updated into the NTP program data. So, we took all TB-07 forms of the study population (notified TB cases from July 2015 to June 2016) and during May and June 2017, we compiled data of all TB-09 forms available in the NTP database for the study period.

### Data Analysis

The collected data was imported, organized, and cleaned into Excel (Microsoft Excel 2016 edition). The cleaned data were checked for accuracy of entry and then analyzed descriptively for the study period for the following process indicators reported in Table 1. Viewing descriptive (statistical) analysis, the categorical variables were presented as counts and proportions (%), and the significance of the statistical test (chi-square) was taken at a *p*-value of <0.05.

**Table 1:** Key definitions of Public–Private Mix (PPM) intervention.

**Indicator**	**Definition**	**Source**
Incident cases	The estimated number of new and relapse cases of Tuberculosis (TB) arising in a given year.	Obtained from the annual WHO global TB report figures of incident TB cases in Pakistan for a given year.
Case notification	The actual number of new and relapsed TB cases reported to WHO for a given year.	Obtained from the annual WHO global TB report figures of notified TB cases in Pakistan for a given year.
Case detection rate	The ratio of the number of reported cases to the number of estimated TB cases in a given year.	Obtained from the annual WHO global TB report figures of TB case detection rate in Pakistan for a given year. Calculated as the number of cases notified divided by the number of estimated cases for that year, expressed as a percentage.
PPM implemented district	The administrative locality/subset unit of a province where the PPM model is being implemented.	Obtained by looking at quarterly case notification report form (TB-07) of a province and noting the names of PPM implemented districts in that province.
PPM coverage	The number of all healthcare facilities that were part of PPM within a PPM implemented district.	By counting and confirming the names of operating PPM facilities in PPM districts mentioned in quarterly case notification report forms (TB-07) from July 2015 to June 2016.
PPM coverage efficiency	The percentage of PPM facilities engaged among the available range of private TB facilities that were not a part of PPM within a PPM implemented district.	By matching the number of PPM TB facilities (NTP Umbrella) with TB facilities operating other than the PPM model (outside NTP Umbrella) in a particular district.
PPM coverage outcome	The mean number of patients with TB notified per PPM TB facility.	Calculated by dividing the PPM-notified cases in total by a total number of participating facilities for a respective PPM model.

## Results

### PPM Implementation Mechanism

The PPM model was operating through a common agreement [memorandum of understanding, (MOU)] between NTP (public arm) and participating private health facility owners (private arm) with relevance to the national TB guidelines. The NTP support mechanism (material supply, financial support, and technical support) to the private provider was also the same and equally distributed in all TB facilities operating through the PPM model (PPM-1, 2, 3, and 4). The NTP support mechanism in all these PPM approaches was provided by the Provincial TB control program (NTP provincial wing) through the district TB control program (NTP district wing). However, the PPM registered cases were notified by private providers to the district TB control program (DTB), then to the Provincial TB control program (PTP), and finally updated into the NTP database.

The four PPM approaches were following a particular set of patient examination, diagnostic, and follow-up procedures. The detailed procedure of the initial presentation of presumptive patients with TB at the aforementioned PPM facilities until their final diagnosis and follow-up plan has been mentioned in Table 2.

**Table 2:** Operational procedure of Public–Private Mix (PPM) models of Tuberculosis (TB) care in Pakistan.

**PPM model**	**Stakeholders involved**	**Operation/patient flow**
Solo general practitioner (GP) model (PPM-1)	There is a formal agreement between a GP preferably a TB specialist (private provider) operating in his private clinic, a district TB coordinator (public sector), and an intermediary NGO responsible for the coordination of public and private providers.	• Presumptive TB patient visits the trained private GP clinics for a regular check-up. • Paramedic staff supporting the GP maintains the patient's record on NTP-provided TB register and provides him with a voucher for performing the diagnostic tests. • The private laboratories on PPM-1 panel perform the tests free of cost on receiving the voucher from visiting patients. • The patient after getting results of all the diagnostic results re-visits the GP for final diagnosis.
Non-Governmental-Organization (NGO) run TB care facility model (PPM-2)	There is a formal agreement between NTP and NGO head along with the willing physician, practicing in NGO clinic	• NGOs' manage TB presumptive and patients through their small hospitals and outpatient clinics with laboratories, providing TB care services. • Paramedic staff supporting the GP in NGO clinics performs the TB case notification and its treatment outcomes on NTP provided recording and reporting (R&R) tool.
Private hospital model (PPM-3)	Medical superintendents of the hospital usually sign the agreement with the NTP team for following the national TB management guidelines.	• PPM-3 facilities operate through trust and large private tertiary care (non-profit) hospitals equipped with TB diagnostic and treatment services and operated by trained staff. • These hospitals offer free-of-charge services to visiting TB patients for TB diagnosis and treatment but charge the patients for non-TB treatment.
Other public sector (Parastatal) model (PPM-4)	Medical superintendents of the parastatal facility are signing authority involved with the NTP team	• PPM-4 model comprises semi-government (autonomous) hospitals with independent administration authorities who provide TB care facilities under one roof essentially to their employees. • It covers healthcare institutions established by organizations working under the administration of the Federal government who do not report to Provincial TB Control Programs (PTP).

### PPM Implemented Districts and Participating Facilities

**Figure 1:**
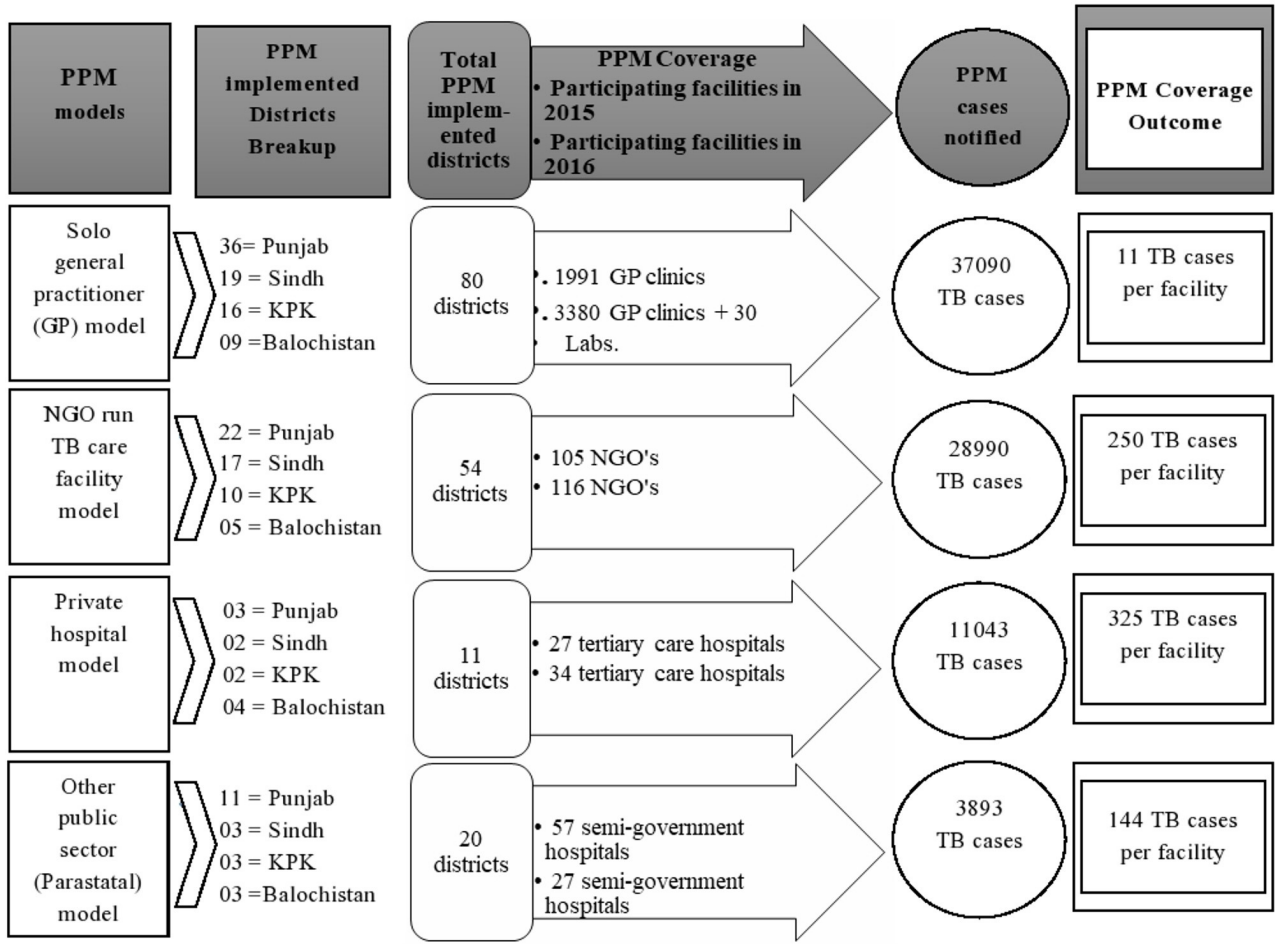
Overview of Public-Private Mix (PPM) model implementation in four provinces of Pakistan from July 2015–June 2016.

Viewing the PPM implementation throughout all four provinces, the PPM model was operationalized in 92 districts in total during the study period (see Supplementary Figure), ranging from 11 districts engagement for the Private Hostel model (PPM-3) to 80 districts implementation in solo GP model (PPM-1). The number of participating PPM facilities within the PPM-implemented district (PPM coverage) showed a varying pattern among all four PPM approaches. The PPM coverage outcome of private hospital facilities was the highest (325 TB cases notified per PPM participating facility) among PPM approaches. The breakup of PPM implemented districts, PPM coverage, and PPM coverage outcome in four provinces is further described in Figure 1.

### PPM TB Case Notification

A total of 327,002 TB cases were notified to NTP from four provinces of Pakistan from mid-2015 to mid-2016. Out of them, 81,016 (25%) cases were contributed by the PPM model. The female-to-male ratio of TB cases is slightly higher among PPM notified cases when compared to non-PPM cases. Table 3 depicts that the PPM TB case notification was considerably higher to non-PPM model in patients aged <15 years (19.4 vs. 8.1%) and >54 years (33.0 vs. 16.5%), respectively but lower in notifying TB cases aging 15–34 years (21.0 vs. 46.6%) and >54 years of age (26.6 vs. 28.8%). The PPM contribution is high (22.5%) when compared to the non-PPM contribution (18.5%) in Sindh province. Chi-square tests for PPM and non-PPM case notification show that a very strong significant difference exists in proportions among compared variables related to gender (*p* < 0.001), age group (*p* < 0.000), and province (*p* < 0.000) involved for the study period.

**Table 3:** Sociodemographic characteristics of patients with tuberculosis (TB) notified by total, non-PPM, and PPM models in Pakistan.

**Variables**	**TB Notification** ** (all forms) *n* (%)**	**Contribution to notification by non-PPM ** **(all forms) *n* (%)**	**Contribution to notification by PPM** ** (all forms) *n* (%)**	***p*-value** ** (PPM vs. non-PPM)**
**Total**	32,7002 (100)	245,986 (75)	81,016 (25)	
**Gender**				
Male	164,355 (50.2)	124,030 (50.4)	40,325 (49.8)	<0.001
Female	162,647 (49.8)	121,956 (49.6)	40,691 (50.2)	
**Age in years**				
<15	35,670 (11.0)	19,958 (8.1)	15,712 (19.4)	<0.000
15–34	131,595 (40.0)	114,605 (46.6)	16,990 (21.0)	
35–54	92,327 (28.0)	70,783 (28.8)	21,544 (26.6)	
>54	67,410 (21.0)	40,640 (16.5)	26,770 (33.0)	
**Province**				
Punjab	210,773 (64.5)	158,837 (64.6)	51,936 (64.1)	<0.000
Sindh	63,648 (19.5)	45,407 (18.5)	18,241 (22.5)	
KPK	43,464 (13.0)	33,350 (13.5)	10,114 (12.5)	
Baluchistan	9,117 (3.0)	8,392 (3.4)	725 (0.9)	

*KPK, khyber pakhtunkhwa; PPM, public–private mix*.

Table 4 shows that out of the total PPM contribution (81,016), a majority (45.8%) of the PPM cases were contributed by solo GP approach, and only (4.8%) cases by Parastatal approach. Province-wise, solo GP clinics notified the highest proportion of patients with TB in Baluchistan (79.4%), Khyber Pakhtunkhwa (68.7%), and Punjab (42.8%), whereas NGOs stood with a maximum (42.1%) PPM contribution in Sindh province. Table 5 indicates that pulmonary cases (79.2%) were notified more than extrapulmonary (20.8%) and clinically diagnosed (43.5%) than bacteriologically diagnosed (32.5%) cases. The run TB care facilities of NGOs were slightly better (81.8%) at diagnosing pulmonary TB. However, the private hospital approach was the only PPM approach that contributed more to bacteriologically confirmed cases than the clinically diagnosed cases (39.9 vs. 32.5%) along with an increased number of relapse cases (5.9%).

**Table 4:** Contribution to national Tuberculosis (TB) case notification by different Public–Private Mix approaches from July 2015 to June 2016.

**Variables**	**Total PPM** ***n* (%)**	**PPM-l^a^** ***n* (%)**	**PPM-2^b^** ***n* (%)**	**PPM-3^c^** ***n* (%)**	**PPM-4^d^** ***n* (%)**
**Total**	81,016 (100)	37,090 (45.8)	28,990 (35.8)	11,043 (13.6)	3,893 (4.8)
**Province**					
Baluchistan	725 (100)	576 (79.4)	79 (10.9)	47 (6.5)	23 (3.2)
KPK	10,114 (100)	6,952 (68.7)	2,509 (24.8)	601 (6.0)	52 (0.5)
Punjab	5,1936 (100)	22,249 (42.8)	18,666 (35.9)	7,461 (14.4)	3,560 (6.9)
Sindh	18,241 (100)	7,313 (40.4)	7,736 (42.1)	2,934 (16.1)	258 (1.4)

a *General practitioner model*,

b *NGO run TB. care facility model*,

c *Private hospital model*,

d *Other public sector (Parastatal) model, KPK, khyber pakhtunkhwa*.

**Table 5:** Disease classification of Tuberculosis (TB) cases notified by different Public–Private Mix approaches from July 2015 to June 2016.

**Variables**	**Total PPM** ***n* (%)**	**PPM-l^a^** ***n* (%)**	**PPM-2^b^** ***n* (%)**	**PPM-3^c^** ***n* (%)**	**PPM-4^d^** ***n* (%)**
**Total**	81,016 (100)	37,090 (100)	28,990 (100)	11,043 (100)	3,893 (100)
**Disease classification**					
Pulmonary	64,193 (79.2)	28,845 (77.7)	23,729 (81.8)	8,653 (78.3)	2,986 (76.6)
Extra Pulmonary	16,823 (20.8)	8,245 (22.3)	5,261 (18.2)	2,390 (21.7)	907 (23.4)
**Type of patients (Pulmonary)**					
Bacteriologically positive	26,332 (32.5)	11,367 (30.6)	9,346 (32.2)	4,402 (39.9)	1,217 (31.2)
Clinically Diagnosed	35,295 (43.5)	16,721 (45.1)	13,356 (46.1)	3,593 (32.5)	1,625 (41.7)
Relapse	2,566 (3.2)	757 (2.0)	1,007 (3.5)	658 (5.9)	144 (3.7)

a *General practitioner model*,

b *NGO run TB. care facility model*,

c *Private hospital model*,

d *Other public sector (Parastatal) model*.

### Treatment Outcomes of PPM-Notified Cases

Referring to Table 6, the overall treatment success rate was recorded (90.6%) for PPM-notified TB cases; ranging from just 46.7% successful treatment in Parastatal facilities to 94.9% success in NGO facilities. The PPM model was more likely to record “treatment completed” than the non-PPM model (69.4 vs. 64.5%) among the favorable outcomes. The unfavorable outcomes (see Table 6) were recorded more for PPM as compared to non-PPM-notified TB cases (9.4 vs. 6%), mostly attributed to Parastatal facilities; this is evident as 53.3% of PPM-4 notified cases were not successfully treated during the study period.

**Table 6:** Accumulated treatment outcomes of Tuberculosis (TB) cases notified to national TB program in Pakistan from July 2015 to June 2016.

**Outcome**	**Total *n*** ** (%)**	**Non-PPM *n* (%)**	**PPM *n*** ** (%)**	**PPM-1 *n*** ** (%)**	**PPM-2 *n*** ** (%)**	**PPM-3 *n*** ** (%)**	**PPM-4 *n*** ** (%)**
Total	327,002 (100)	245,986 (100)	81,016 (100)	37,090 (100)	28,990 (100)	11,043 (100)	3,893 (100)
Cure	89,808 (27.5)	72,655 (29.5)	17,153 (21.2)	8,134 (21.9)	6,531 (22.5)	2,309 (20.9)	179 (4.6)
Tx* Complete	214,851 (65.7)	158,642 (64.5)	56,209 (69.4)	26,854 (72.4)	20,990 (72.4)	6,725 (60.9)	1,640 (42.1)
Subtotal Success	304,659 (93.2)	231,297 (94.0)	73,362 (90.6)	34,988 (94.3)	27,521 (94.9)	9,034 (81.8)	1,819 (46.7)
Failed	1,414 (0.4)	820 (0.3)	594 (0.7)	203 (0.5)	236 (0.8)	153 (1.4)	2 (0.0)
Died	4,313 (1.3)	3,069 (1.2)	1,244 (1.5)	646 (1.8)	219 (0.8)	360 (3.2)	19 (0.5)
LTFU	9,571 (2.9)	7,697 (3.1)	1,874 (2.3)	426 (1.1)	350 (1.2)	993 (9.0)	105 (2.7)
Not evaluated	4,148 (1.3)	3,014 (1.2)	1,134 (1.4)	343 (1.0)	275 (1.0)	474 (4.3)	42 (1.1)
Record not found	2,897 (0.9)	89 (0.0)	2,808 (3.5)	484 (1.3)	389 (1.3)	29 (0.3)	1,906 (49.0)
Subtotal unfavorable	22,343 (6.8)	14,689 (6.0)	7,654 (9.4)	2,102 (5.7)	1,469 (5.1)	2,009 (18.2)	2,074 (53.3)
LTFU/NE/ RNF	16,616 (5.0)	10,800 (4.4)	5,816 (7.1)	1,253 (3.4)	1,014 (3.5)	1,496 (13.5)	2,053 (53.0)
Failed/died	5,727 (1.8)	3,889 (1.6)	1,838 (2.3)	849 (2.3)	455 (1.6)	513 (4.6)	21 (0.3)

^a^General practitioner model, ^b^NGO run TB care facility model, ^c^Private hospital model, ^d^Other public sector (Parastatal) model; Tx*, treatment; LTFU, loss to follow up; NE, not evaluated; RNF, record not found.

## Discussion

This study was the first large-scale study to assess the individual contribution of different approaches of the PPM model in Pakistan after its cohesive implementation at the national level. Overall, the PPM model contributed to the TB case notification (by 25%) in program data of NTP between July 2015 and June 2016, suggesting that more TB cases can be notified utilizing PPM intervention in Pakistan. This finding is considerably consistent with countries that have prioritized the PPM model for an increasing trend in TB case notification to their national TB control programs (5). Of the WHO-reported countries contributing between 5 and 56% to TB notification through PPM (18), our study was of value with a 25% contribution to the national database.

Disaggregating TB case notification by gender shows that PPM notified more TB cases in females than males, which is contrary to the non-PPM proportion of male TB cases exceeding female cases. PPM and non-PPM gender patterns in the notification are, respectively, in accord with previous study trends in sex-specific TB case notification in western and eastern provinces of Pakistan over 10 years (19). In terms of individual age groups involved, the PPM model is most likely to identify pediatric and elderly aged TB cases in comparison to the non-PPM model. The enhanced contribution of PPM to the pediatric age group may be attributed to the decision of NTP to engage PPM as one of the five key initiatives to detect missed childhood TB cases and minimize delayed diagnosis of these TB cases (20). Elderly aged patients with TB may prefer PPM facilities because of more access, response, and individualized option in comparison to non-PPM facilities (21), which might not be the case for the preference of adult-age patients with TB for initiating TB care at these facilities. Hence, the PPM model was contributing to a smaller number of young adult and middle-age TB cases. So, this varying pattern of health-seeking among all four patient age groups involved in this study reveals that different age group patients with TB tend to seek and initiate TB care differently (i.e., some prefer PPM, whereas others non-PPM) in the Pakistani community.

From the province viewpoint, the PPM model is more likely to identify TB cases in Sindh when compared to the non-PPM model. This might well be due to the low utilization of public sector health services by the population due to the non-availability of staff and medicines and thus seeking TB care more from private providers in Sindh (22). PPM findings of less contribution in Khyber Pakhtunkhwa and Baluchistan might be attributed to population preference in these two provinces to access public (government) sector hospitals more than private hospitals due to their financial constraints (23). However, qualitative studies can be performed in the future to explore the reasons for less contribution of PPM in the province of Punjab in comparison to non-PPM TB case notification.

Among PPM approaches, the solo GP model contributed the highest percentage of total PPM cases in Punjab, Khyber Pakhtunkhwa, and Baluchistan (see Table 2). This might well be due to the GP model engagement in an extensive number of PPM implemented districts (80 out of total 92 districts), and improved PPM coverage: as 1991 GP clinics in 2015 were almost doubled to 3,380 GP clinics and 33 private laboratories in 2016, whereas the number of health facilities for the remaining PPM approaches remained nearly constant or dropped throughout the study period (see Figure 1). The total solo GP model contribution of 46% in this study is in contrast with the previous study in 2009, which documents only 5% PPM-1 contribution to total PPM cases mainly due to PPM-1 operational issues and limited health facilities implementation (14), that seems to be largely resolved during our study. However, the lowest treatment coverage outcome of GP facilities (11 TB cases per facility) in comparison to the remaining PPM approaches indicates that only a few GPs might have been actively involved in enrolling patients with TB at their clinics. Despite the hugely engaged network of GP clinics in four provinces, this low level of GP commitment in TB patient enrolment is consistent with experiences learned from PPM implemented GP clinics in six towns of Karachi (24).

The PPM-implemented districts and PPM coverage may not be the sole indicators for improved PPM TB case notification when we compare PPM-notified cases of GP and NGO facilities in Sindh province. That is, with a slightly different number of PPM-implemented districts for both GP and NGO approaches in Sindh (19 vs. 17 districts), and an overall reduced range of coverage of NGOs in comparison to the latter (116 vs. 3,380 facilities), the approach of NGOs still was the marginally largest PPM contributor in Sindh. First, this might be due to the enhancement in PPM coverage efficiency of the NGO model in the province, as 61 out of 62 available NGO facilities offering TB services in Sindh were working under the umbrella of NTP during the study period. The second possible reason might be the improvement in PPM coverage outcome of NGO facilities in comparison to GP facilities (250 cases vs. 11 cases notified per participating facility). This enhanced NGO cases per facility finding (PPM coverage outcome) were also consistent with large-scale partnerships between NGO-led model and NTP for improving TB services for the high-risk population in India (25). Hence, the improved PPM coverage outcome of NGO facilities in comparison to GP facilities in Sindh points out another important PPM indicator, that is, among two PPM models that have nearly the same implementation in districts, the model having improved PPM coverage outcome can likely contribute more PPM cases than the second model with reduced PPM coverage outcome.

Moving away from the concept of comparing PPM approaches with an almost equal number of PPM-implemented districts but having different PPM coverage outcomes toward PPM approaches with an unequal distribution of PPM-implemented districts and different PPM coverage provides another important PPM insight. The private hospital approach (PPM-3) is adopted with the least number of PPM-implemented districts in the study period (i.e., 11 out of 92 PPM districts), but its contribution in terms of PPM coverage outcome is highest (325 cases notified per participating facility) in comparison to the remaining PPM approaches (PPM-1 = 11, PPM-2 = 250, PPM-4 = 144 cases per facility). This shows that a PPM approach even with the lowest implementation in a district (such as PPM-3) can have an improved rank in contributing PPM cases per facility in comparison to the PPM coverage outcome of other models with a much higher number of PPM-implemented districts involved. This finding also indicates that Private hospital facilities might be a useful resource in terms of the number of PPM cases initiating TB care per PPM facility but needs to be scaled up for overall improvement in the remaining PPM indicators for the enrolled patients. This whole discussion of different PPM indicators (PPM-implemented district, PPM coverage, PPM coverage efficiency, and PPM coverage outcome) is reflective of their importance at various steps of scaling up the PPM model in future studies in Pakistan.

Viewing the remaining socio-demographics of PPM-notified cases, among the pulmonary cases, clinically diagnosed patients were notified more than bacteriologically confirmed ones; mostly attributed by NGOs, closely followed by GPs. This indicates that both NGOs and GP facilities were associated with over-reliance on radiography, suggestive disease histology, and the under-use of sputum smear microscopy for diagnosis. This diagnosis pattern of GP clinics is consistent with clinicians in other countries (26), suggesting a careful review of recruiting and training private practitioners in PPM-DOTS in Pakistan (27). While for NGO-run TB care facilities, future research can be conducted to explore the possible reasons for over-reliance. However, the private hospital approach remains the sole PPM approach that was not only good in detecting bacteriologically confirmed among pulmonary cases but also slightly better in notifying patients previously treated, and now diagnosed with a recurrent episode of TB.

Among notified patients who started treatment in PPM facilities, the treatment success rate (90.6%) was considerably higher than the WHO-recommended target of 85% (28). This finding also supported previous PPM studies in Pakistan showing that the PPM model is not limited to contributing to TB case findings but also maintains a good treatment success (24, 27). Among PPM approaches, GP and NGO facilities achieve the same outcomes as non-PPM, with treatment success of 94–95% and only 3–4% not evaluated or lost; private hospitals achieve 82% success because of a total of 14% not evaluated or lost; Parastatals are unable to follow more than half of cases, and therefore achieve a success rate of just 47%, the number of participating facilities fell from 57 in 2015 to 31 in 2016, and they contributed only 1.1% of case notifications in these four provinces from mid-2015 to mid-2016. Private hospital facilities may have slightly higher rates of failure/death (4.65 vs. 1.8% overall) but this may well be due to more complicated cases presenting to hospitals. “Loss to follow-up” and “Not evaluated” were also recorded more in private hospitals. This default for the hospital was consistent with PPM findings in Indonesia (29).

Out of 81,016 patients with TB, the outcome record of 2,808 PPM cases was found missing; these cases were notified (on TB-07) but their outcome record (on TB-09) was not available, mostly attributed to Parastatals. This was considerably high as compared to non-PPM contribution (3.5 vs. 0.0%) and reflects a gap between notification and recording treatment outcome in the PPM model particularly, within Parastatal facilities. This gap can be covered by developing PPM mechanisms for better documentation and patient follow-up (30). Combining the percentage contribution of notified TB patient's outcomes with “loss to follow-up,” “not evaluated,” and “record not found” PPM contribution is still high than non-PPM (7.1 vs. 4.4%) (see Table 6). This finding can be reflective of the need to think beyond PPM-DOTS expansion and start focusing on the quality of TB care provided in PPM facilities (31). The enhancement in quality of TB care in the PPM model can involve implementing PPM approaches based on socio-cultural dynamics and processes (32), introducing national electronic-case-based surveillance for TB in PPM facilities (5), and involving and training remaining TB stakeholders who often serve as the first point of contact for TB care such as pharmacists (33–37), nurses (38, 39), and traditional healers (40), which are largely neglected as a part of the healthcare team within PPM model in Pakistan.

### Strengths

The first strength of the PPM intervention study (different approaches of conventional PPM model), is that the data was collected from routinely maintained program data, so the findings are likely to reflect the program realities. Second, the study population has a large sample size, our estimate is likely to be precise. Third, the STROBE guidelines were followed for study design, methodology, and reporting of outcomes which minimizes the risk of methodological biases (41). Fourth, the study provides evidence and a descriptive overview of total TB case notification (PPM and non-PPM) from all TB treatment facilities available for 97% Pakistani DOTS population residing in four provinces in the study period.

### Limitations

First: the PPM intervention study was limited to the quantitative assessment of different PPM approaches. Qualitatively, it could not explore the reasons either for performance perspectives of the model's stakeholders and patients undergoing treatment in PPM facilities or variation of PPM contractual arrangements in different provinces and areas of Pakistan. Second: a record review was done and the reliability of routinely collected data cannot be assured. Third: the figures were extracted using aggregate data which is expected to have minor fluctuations as compared to individual facility data. Fourth: the study could not include the PPM case notification of the remaining 3% Pakistani population due to ethical approval constraints by NTP. Lastly, the aggregate PPM data lacks the segregation of participating GPs' working either in Government as well-private TB facilities during morning and evening timings or practicing solely in the private health sector.

## Conclusions

The PPM intervention is contributing substantially to the national TB case notification in possibly all the age groups, gender, and provinces, so the PPM model implementation is requisite to detect missing cases and end the TB epidemic in the Pakistani healthcare system. Based on the evidence presented for different approaches of the cohesively implemented PPM model in this study, we conclude that GPs, NGOs, and Private hospital facilities should be scaled up, but Parastatal facilities need to be substantially reformed.

## Recommendations

Among PPM approaches (PPM-1,-2,-3, and−4), GP facilities scale-up should be focused more toward their PPM coverage outcome as well as revising the PPM-1 strategies for inclusion of GPs' exclusively practicing TB care in the private sector, while NGO facilities for an increase in their PPM coverage, and Private hospitals in terms of enhancement in PPM coverage efficiency, PPM implemented districts, along with extending the treatment success rate up to 85%. Reforms in Parastatal facilities should not only be aimed at upgrading the Parastatal contribution toward PPM TB case notification but also improving their follow-up mechanisms to avoid the default of Parastatal notified TB cases as well as retaining the number of Parastatal facilities offering TB services.

Moreover, a strong PPM infrastructure for TB control is vital for a country where the private sector in its current state (i.e., cohesively implemented PPM model), contributes considerably to the notification of missing TB cases. To reach out the missing TB cases further though PPM model, the mobile, robust, and systematic inclusion of the remaining nine private TB providers (such as pharmacists, nurses, philanthropists, chemists, community leaders, religious leaders, researchers, insurance agencies, and mineral industries) in the Pakistani PPM program are needed. This can be achieved either through their recognized participation as a part of the national TB healthcare team, legislating the mandatory TB case notification act in the remaining provinces of KPK, Sindh, and Balochistan, or incentivizing their TB referral services according to national TB guidelines in the form of an MOU. In the future, the engagement of all private providers of TB care will not only provide equity of access to an extended number of the patients seeking care for TB treatment in Pakistan but can also extend the current PPM contribution (from 25 to 56%) to the national TB database.

## Data Availability Statement

The original contributions presented in the study are included in the article/Supplementary Material, further inquiries can be directed to the corresponding author.

## Ethics Statement

The ethical approval for the conduct of this project was obtained from the Advanced Studies and Research Board, Quaid-i-Azam University Islamabad, and Research Unit, NTP Pakistan. Written informed consent for participation was not required for this study in accordance with the national legislation and the institutional requirements.

## Author Contributions

WU conceived and designed the study, collected, interpreted, and analyzed the data, drafted the first version of the paper, and revised the final manuscript. MH coordinated for data collection and provided input into the data interpretation. AW coordinated for data collection, provided input into the data interpretation, and assisted in revising the manuscript. AY provided advice on data analysis and interpretation. RF assisted in the conceptual phase and study design, reviewed the first draft, and assisted in revising the final manuscript. GK assisted in the conceptual phase and study design, reviewed the first draft, and provided approval to the final manuscript. All authors contributed to the article and approved the submitted version.

## Conflict of Interest

The authors declare that the research was conducted in the absence of any commercial or financial relationships that could be construed as a potential conflict of interest.

## Publisher's Note

All claims expressed in this article are solely those of the authors and do not necessarily represent those of their affiliated organizations, or those of the publisher, the editors and the reviewers. Any product that may be evaluated in this article, or claim that may be made by its manufacturer, is not guaranteed or endorsed by the publisher.
