# Supplementary material for: Public–Private Mix Models of Tuberculosis Care in Pakistan: A High-Burden Country Perspective
Source: Front Public Health. 2021 Aug 10;9:703631. doi: 10.3389/fpubh.2021.703631 (PMC8383070; doi:10.3389/fpubh.2021.703631)
Supplement: Supplementary file 1 [file Table_1.DOCX]

**Figure:** Map of PPM implemented districts in four provinces of Pakistan


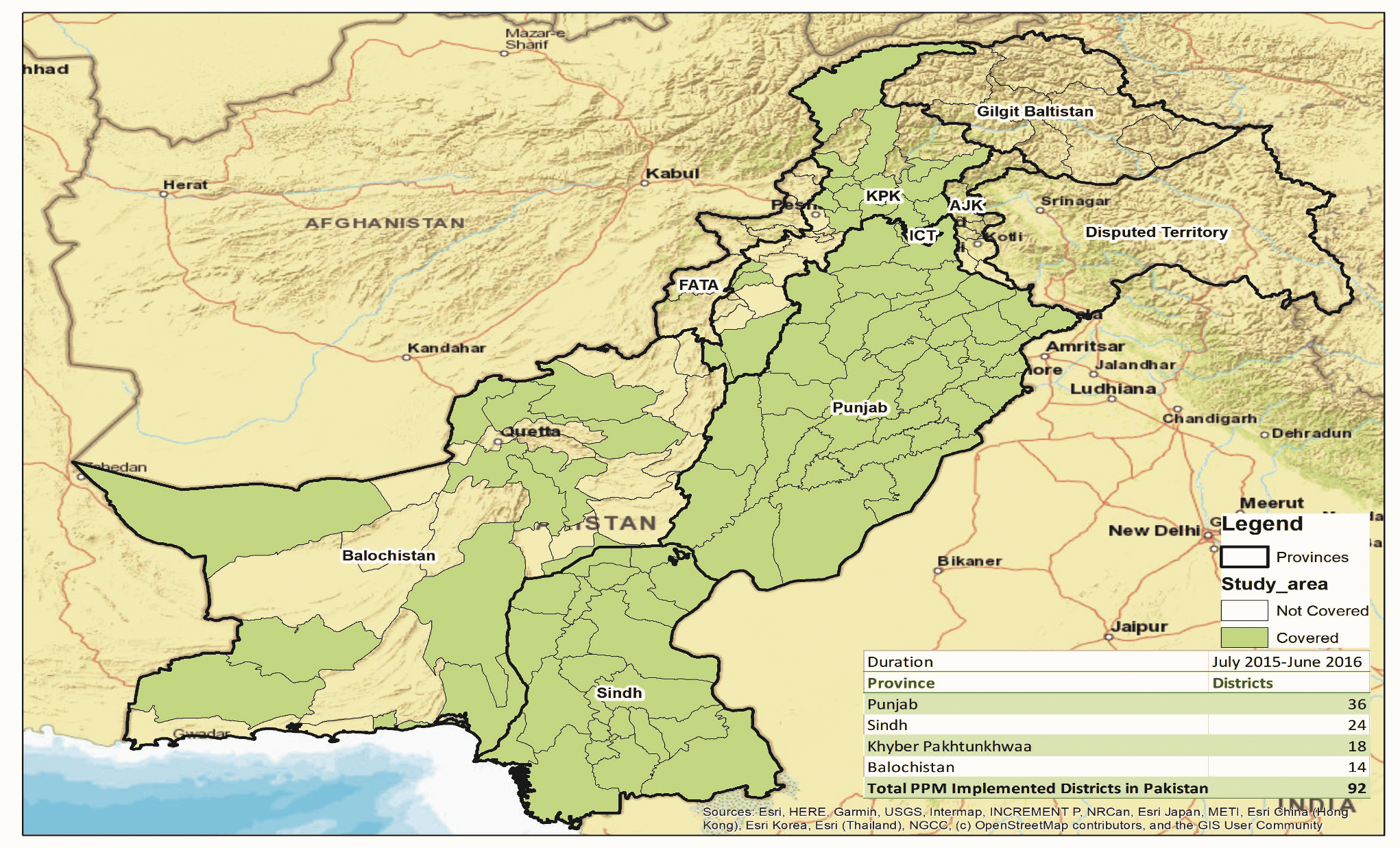


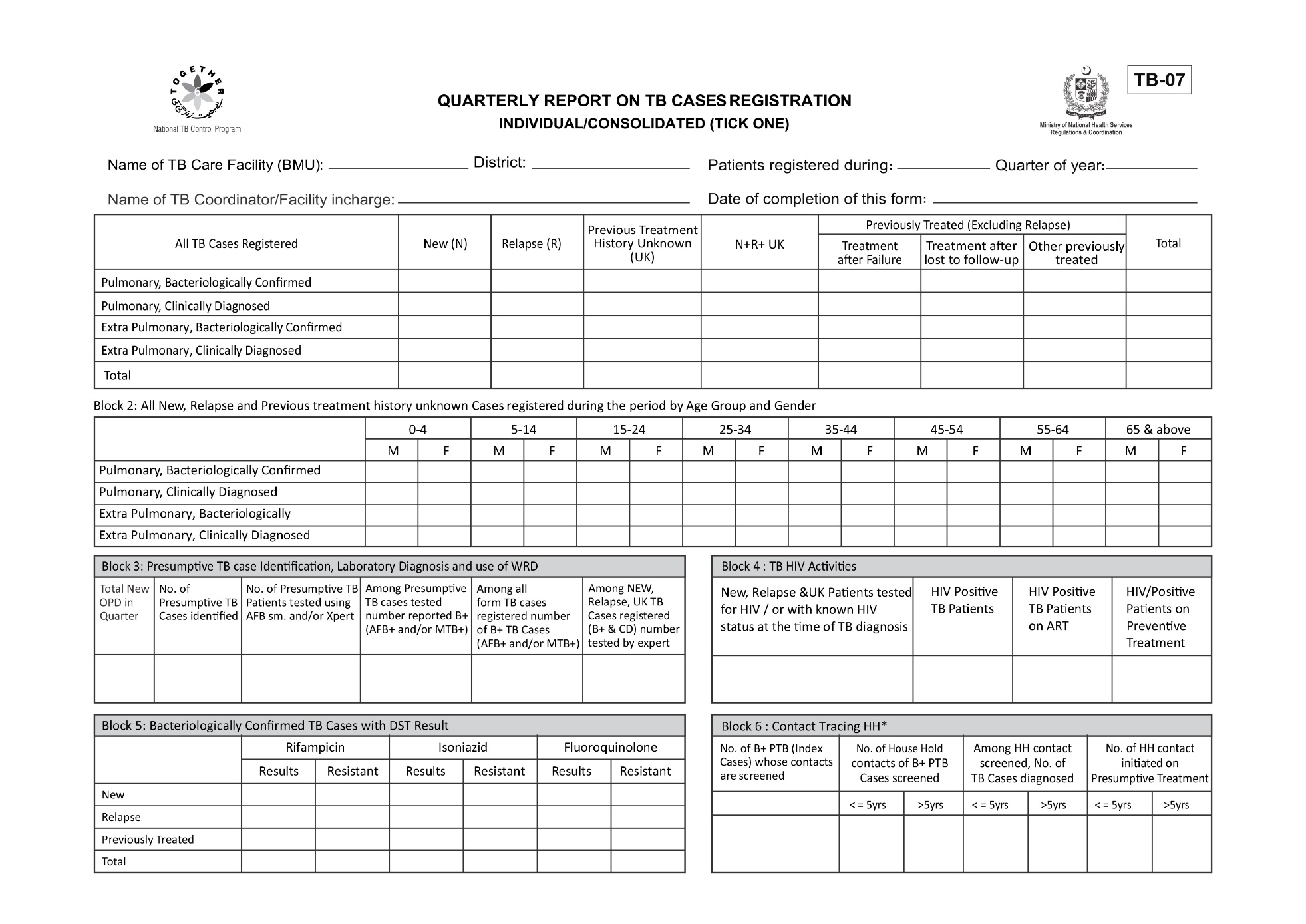
**DATA COLLECTION FORMS (TB-07 & TB-09)**

**
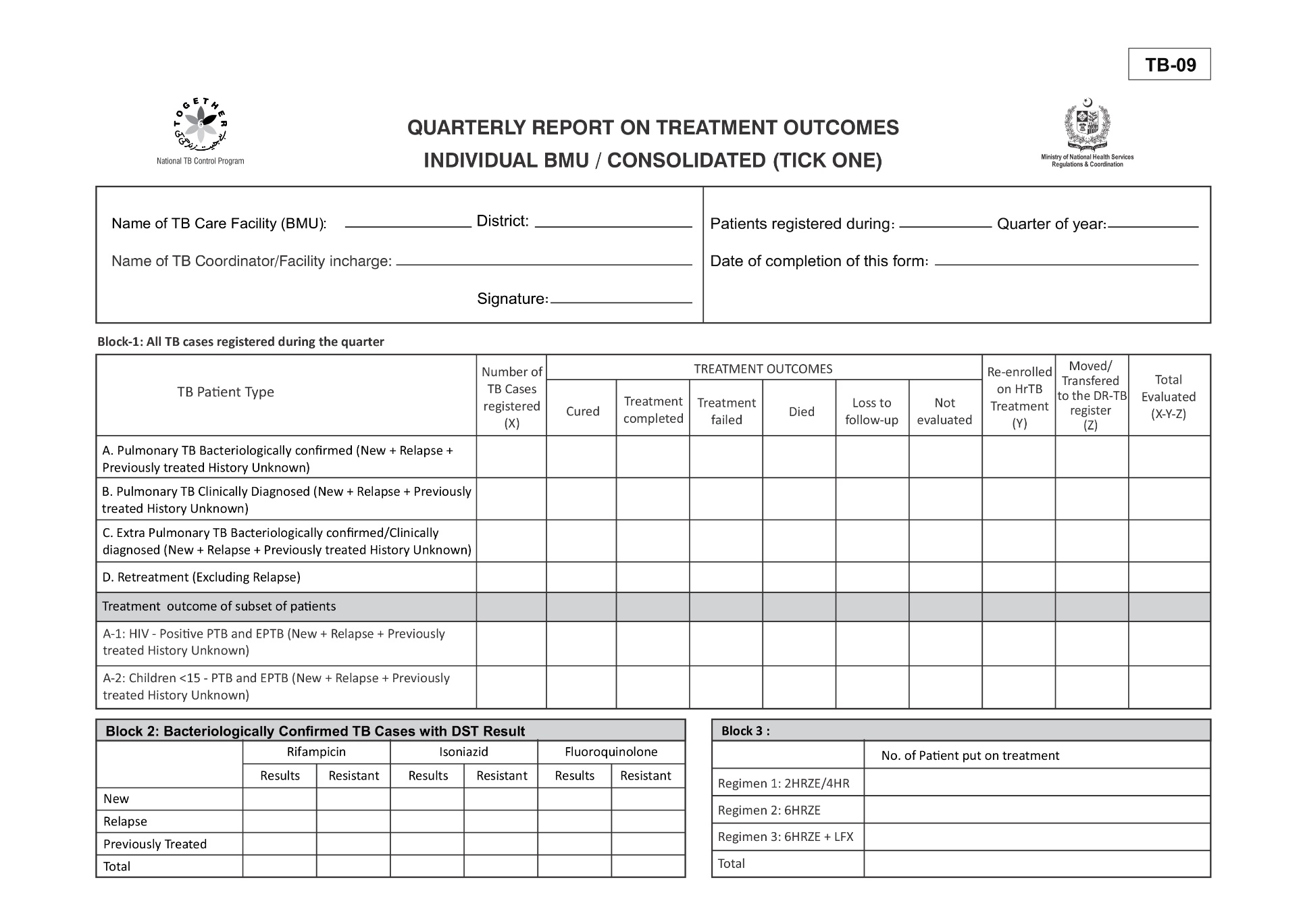
**
